# Creating new functional circuits for action via brain-machine interfaces

**DOI:** 10.3389/fncom.2013.00157

**Published:** 2013-11-05

**Authors:** Amy L. Orsborn, Jose M. Carmena

**Affiliations:** ^1^UC Berkeley - UCSF Joint Graduate Program in Bioengineering, University of California BerkeleyBerkeley, CA, USA; ^2^Department of Electrical Engineering and Computer Science, University of California BerkeleyBerkeley, CA, USA; ^3^Helen Wills Neuroscience Institute, University of California BerkeleyBerkeley, CA, USA

**Keywords:** brain-machine interfaces, motor learning, neural plasticity, volitional control, sensorimotor systems

## Abstract

Brain-machine interfaces (BMIs) are an emerging technology with great promise for developing restorative therapies for those with disabilities. BMIs also create novel, well-defined functional circuits for action that are distinct from the natural sensorimotor apparatus. Closed-loop control of BMI systems can also actively engage learning and adaptation. These properties make BMIs uniquely suited to study learning of motor and non-physical, abstract skills. Recent work used motor BMIs to shed light on the neural representations of skill formation and motor adaptation. Emerging work in sensory BMIs, and other novel interface systems, also highlight the promise of using BMI systems to study fundamental questions in learning and sensorimotor control. This paper outlines the interpretation of BMIs as novel closed-loop systems and the benefits of these systems for studying learning. We review BMI learning studies, their relation to motor control, and propose future directions for this nascent field. Understanding learning in BMIs may both elucidate mechanisms of natural motor and abstract skill learning, and aid in developing the next generation of neuroprostheses.

Recent technological advances have made it possible to directly connect brains with machines. Recorded neural activity can be used to control external devices in real-time, and neural stimulation can be applied based on external events to convey information into the brain. These brain-machine interfaces (BMIs) have a wide range of potential applications, including rehabilitative and restorative therapies for patients with neurological deficits. Cochlear implants, for instance, are widely used to restore hearing to patients with severe hearing loss. Recently, there have been demonstrations of BMIs used to restore movement in paralyzed humans by using neural signals to control external devices (Hochberg et al., 2006, 2012; Collinger et al., 2012).

Much as there are many potential applications for BMI technology, there are a variety of possible implementations. BMIs can be used to replace motor or sensory systems, or both simultaneously. Motor (efferent) BMIs use recorded neural activity to control external devices, while sensory (afferent) BMIs use neural stimulation to transmit information to the brain. Many different types of neural signals can be used for efferent control including electroencephalography (EEG), electrocorticography (ECoG), local-field potentials (LFPs), or single- and multi-unit action potentials. Neural activity has been successfully used to control a variety of devices in real time, including virtual objects (Serruya et al., 2002; Taylor et al., 2002; Carmena et al., 2003; Leuthardt et al., 2004; Wolpaw and McFarland, 2004; Hochberg et al., 2006; Jarosiewicz et al., 2008; Kim et al., 2008; Schalk et al., 2008; Ganguly and Carmena, 2009; Suminski et al., 2010; O’Doherty et al., 2011; Gilja et al., 2012; Engelhard et al., 2013; Rouse et al., 2013; Wander et al., 2013), robots (Carmena et al., 2003; Taylor et al., 2003; Millán et al., 2004; Velliste et al., 2008; Collinger et al., 2012; Hochberg et al., 2012), wheelchairs (Millán et al., 2009), or to drive movements of the user’s body via muscle stimulation (Moritz et al., 2008; Ethier et al., 2012). Similarly, neural stimulation for sensory BMIs can be implemented using electrical approaches, such as intracortical microelectrode stimulation (ICMS), or via optogenetic methods. Stimulation at different levels of the central nervous system (CNS) has been used to convey auditory (Wilson et al., 1991), visual (Weiland and Humayun, 2008; Tehovnik et al., 2009), tactile (Romo et al., 2000; O’Doherty et al., 2011; Venkatraman and Carmena, 2011; Berg et al., 2013), and proprioceptive (London et al., 2008) feedback to users. Recent work also shows that sensory and motor BMIs can be combined (O’Doherty et al., 2011), which holds great promise for restoring function to paralyzed individuals lacking somatosensory feedback.

Decades of work in BMIs has produced impressive demonstrations, but also reveals that interfacing the brain with machinery is not a simple matter of restoring broken connections. Indeed, these interfaces create new systems that can engage learning and adaptation (Fetz, 2007). Interestingly, BMIs are distinct from the natural sensorimotor apparatus, yet still involve select components of the CNS. Understanding these unique systems may be particularly important for engineering development of successful neuroprosthetic systems (Ganguly and Carmena, 2009; Gilja et al., 2012). They may also provide unique advantages for exploring fundamental questions in neuroscience. BMIs provide scientists a rare opportunity to create novel, well-defined functional circuits that are separate from, but parallel to, their natural counterparts.

BMIs may be particularly useful for studying questions of motor learning and skill formation. While studying the natural sensorimotor system has revealed significant insights, many questions about the neural mechanisms of skill learning remain (Wolpert et al., 2011). For instance, how are learned skills stored in the brain, and what brain areas facilitate their formation and recall? What are the neural underpinnings of performance optimization and refinement? BMIs create novel, functional circuits for action and/or sensation that can be used to study skill learning *de novo* and subsequent adaptation. Because these systems are defined by the experimenter, they may also reduce ambiguities inherent in neurophysiological motor learning studies caused by the complexities of the highly distributed natural motor control system. BMIs define a simpler, known mapping between neural activity and behavior, allowing for careful study of learning-related changes in neurons directly and in-directly contributing to behavior. BMI systems can be more readily interrogated and manipulated by the experimenter to provide new insights into the neurophysiological basis of learning.

Interestingly, BMIs can also be used to define systems that operate irrespective of the natural sensorimotor apparatus. Indeed, motor BMIs can be operated without movement (Taylor et al., 2002; Carmena et al., 2003; Ganguly and Carmena, 2009; Koralek et al., 2012). This property of BMIs might be useful for studying learning of more abstract, cognitive skills. Very little is known about how we acquire skills independent of movement, like solving puzzles. Closed-loop BMIs can be used to define new input-output relationships, or transforms, for the CNS to learn and solve irrespective of the natural sensorimotor system. Moreover, selection of different neural inputs for BMI could be used to study learning in a variety of brain areas and systems. We refer to skill learning in these novel systems controlled irrespective of movement as *neuroprosthetic skills*. Studying these neuroprosthetic skills may be particularly useful for understanding how abstract, non-physical skills are learned, and their neural representations.

Recent work in BMIs demonstrates the potential utility of this paradigm for studying learning, with a growing number of papers providing evidence about the neural mechanisms of adaptation and skill consolidation. Emerging work in sensory BMIs and other closed-loop interface systems also show great promise. Here, we discuss the interpretation of BMIs as novel closed-loop systems partially removed from the CNS. We review the key aspects of these systems that make them uniquely suited to motor learning studies, summarize work demonstrating their potential, and explore future avenues of research.

## BMIs create novel closed-loop control systems

**Figure 1:**
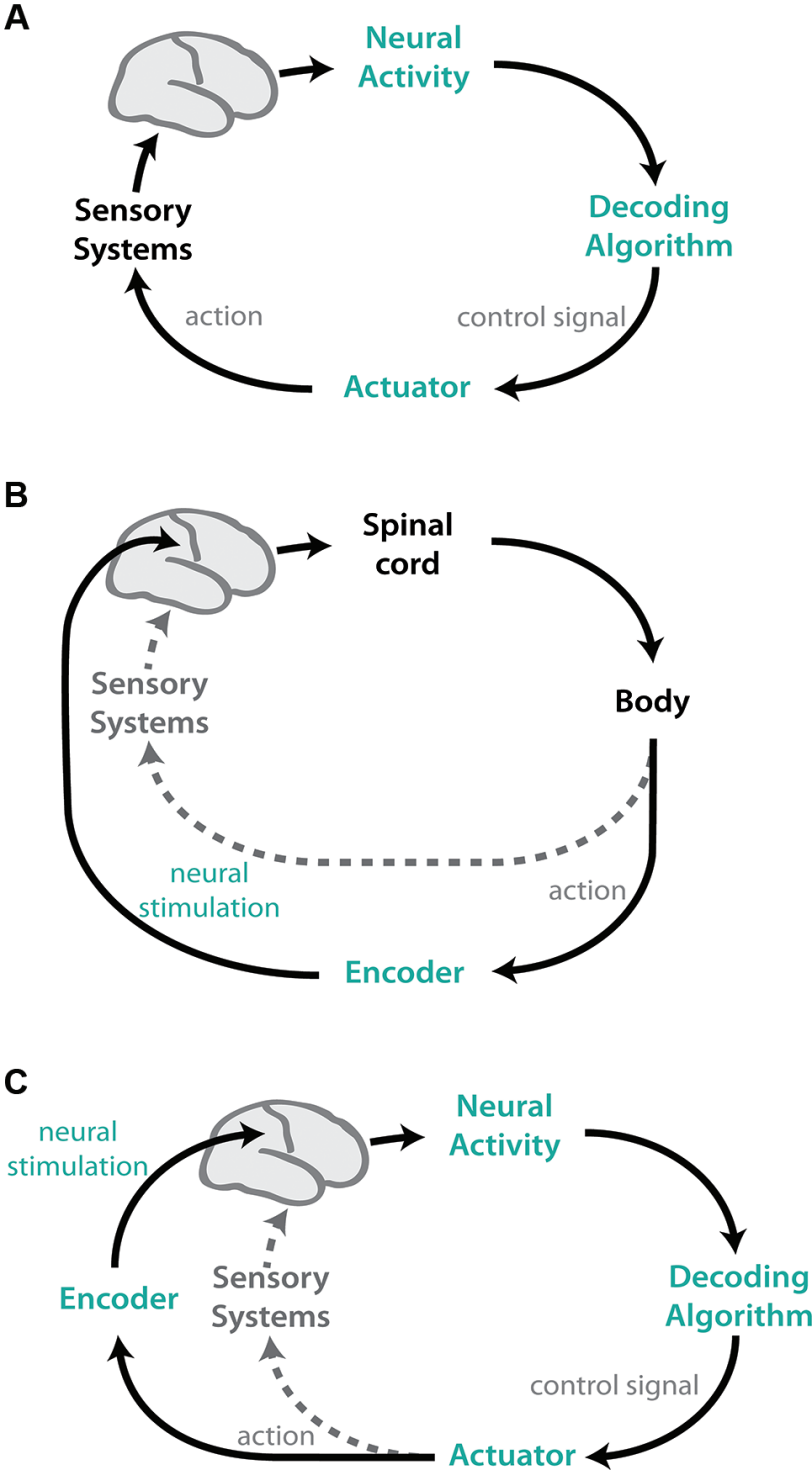
**Schematic representations of BMI systems.** Components of the natural CNS are shown in black/grey; artificial, experimenter-controlled elements are colored. **(A)** Motor (efferent) BMIs map recorded neural activity into control signals for a device via a decoding algorithm. These systems typically use natural sensory systems, such as vision, to provide feedback to the user, creating a closed-loop system. **(B)** Sensory (afferent) BMIs use the natural motor apparatus to perform actions, but close the control loop using feedback conveyed via neural stimulation. Environmental variables are encoded into patterns of stimulation delivered to select brain regions. The artificial feedback can also be combined with natural sensory stimuli (grey dotted). **(C)** Afferent and efferent BMIs can be combined, where actions are decoded from neural activity and feedback is provided via encoded neural stimulation. Again, the artificial sensory feedback can be combined with natural sensory systems (grey dotted).

Despite variability in implementation, at their core, all sensorimotor neuroprostheses are simple closed-loop control systems (Figure 1). In motor BMIs, neural activity is recorded and an algorithm, “the decoder”, is used to map the neural activity into a control signal to move the actuator. Feedback is provided to the user to create a closed-loop control system (Figure 1A). These systems typically use natural sensory systems, such as vision and/or audition, for feedback. Sensory BMI systems, in contrast, use algorithms to “encode” relevant information into neural stimulation patterns that are delivered to the brain (Figure 1B), and actions are implemented using the natural motor apparatus. This neural stimulation can also be combined with other native sensory feedback. These two approaches can be combined such that actions are implemented by a BMI controller, and feedback is provided using neural stimulation (Figure 1C).

The closed-loop nature of BMI systems is essential to their operation and makes them a particularly useful tool for studying learning. The feedback in BMI systems allow users to modify their behavior to achieve desired goals. Many studies show that rats, non-human primates and humans can learn to volitionally control neural activity using biofeedback at the level of single-unit action-potentials (Fetz, 1969, 2007; Fetz and Finocchio, 1971, 1975; Chapin et al., 1999; Gage et al., 2005; Cerf et al., 2010; Moritz and Fetz, 2011; Koralek et al., 2012), local field potentials (Engelhard et al., 2013; Flint et al., 2013), ECoG (Leuthardt et al., 2004; Schalk et al., 2008; Rouse et al., 2013), and EEG (reviewed in Wolpaw et al., 2002; McFarland and Wolpaw, 2011). Volitional control can also decouple single-unit activity from its typical functional roles (Fetz and Finocchio, 1971, 1975). Increasing research shows these volitional control plays a role in closed-loop BMI operation. For instance, subjects can learn to modulate neural activity in order to improve efferent BMI performance for a given decoder (Ganguly and Carmena, 2009).

It is also crucial to understand BMI systems’ relation to the natural sensorimotor system. Consider motor BMIs for upper limb reaching. Natural arm movements are orchestrated by a host of brain areas, the spinal cord, and limb biomechanics; and somatosensory, proprioceptive and visual feedback play critical roles in the control. In BMI, subjects typically control an artificial device via visual observation alone, whose movements are governed by the activity of only a small subset of neurons in motor cortical areas (e.g., primary motor cortex). While BMIs can engage other cortical and subcortical areas (Ganguly et al., 2011; Koralek et al., 2012; Wander et al., 2013), the relationship between movement and neural activity imposed in BMI differs significantly from that of natural movements. These BMI systems may be able to replace motor function, but do so by creating a new control system that is distinct from natural arm movements. Yet, this novel system still incorporates elements of the natural system. For instance, motor BMIs (e.g., driven by activity from primary motor cortex) and arm movements both engage motor cortical areas. The control of BMI systems may, then, share key similarities to the control of natural movements. Research does suggest strong connections between natural motor learning and learning BMIs, as reviewed in Green and Kalaska (2011); Jackson and Fetz (2011) and the discussions below. BMI systems are separate from, but parallel to, the native functions they imitate.

Historically, work in BMIs have not fully appreciated the novel aspects of closed-loop BMI systems^1^. Many have focused on mimicking the natural system (reviewed in Jackson and Fetz, 2011), developing decoding algorithms to predict limb movements or motor intentions from neural activity. However, increasing research shows that a decoder’s prediction power does not necessarily translate to improved closed-loop performance (Koyama et al., 2009; Ganguly and Carmena, 2010; Cunningham et al., 2011). This has led some to re-examine BMI systems and the underlying assumptions of biomimetic approaches (Jackson and Fetz, 2011; Gilja et al., 2012). Recent work incorporating closed-loop perspectives shows great promise for improving BMI performance (Gilja et al., 2012). Understanding BMIs as closed-loop systems distinct from their natural counterparts may be essential for applications of BMI technology. Moreover, this insight opens up many possibilities for using it as a tool to study learning.

One of the most interesting and potentially valuable aspects of BMIs is that they allow experimenters to fully define functional circuits for action. The control system created by closed-loop BMIs are specified by the experimenter. Efferent BMIs define: (1) the neural activity used for control—i.e., the system input, (2) the mapping of how neural activity influences performance, manipulated via the decoder, (3) the variables controlled by the brain, and (4) the types of feedback provided to the user. Similarly precise control is available for afferent BMI systems, which stipulate what information is transmitted, how, and to what brain areas. This control also allows for more complete observation and analysis of the system during learning. For instance, neurophysiological studies during motor adaptation can feasibly monitor a small subset of the neurons within the highly distributed motor system (Wise et al., 1998; Gandolfo et al., 2000; Li et al., 2001; Paz et al., 2003; Padoa-Schioppa et al., 2004; Paz and Vaadia, 2004). Though informative, this captures only a portion of neural learning mechanisms. Moreover, the direct relationship between the activity of individual neurons in motor cortical areas and behavior is also still a topic of significant debate (e.g., reviewed in Shenoy et al., 2013), complicating mechanistic interpretations of neural activity changes with learning (Jarosiewicz et al., 2008). BMI systems artificially constrain the neural input and/or output, and in doing so provide full knowledge of the input-output mapping governing the system behavior. This may allow for more direct assessments of learning-related changes. The ability to create simplified control circuits could highlight principles and mechanisms that may be less clear in a more complex control system. However, leveraging the full potential of this aspect of BMIs also requires understanding how BMI relates to the natural motor system. We return to this critical question in the discussion below.

Finally, by creating new functional circuits, BMIs define novel tasks for the CNS to learn. Many classic sensorimotor learning paradigms apply perturbations to the natural motor system—for example, in the form of forces (e.g., Shadmehr and Mussa-Ivaldi, 1994) or visuomotor transformations (e.g., Krakauer et al., 2000)—and study how the nervous system learns these modifications (recently reviewed in Wolpert et al., 2011). How subjects learn these tasks may be shaped by prior experience, since these tasks modify the subjects’ natural motor repertoire (Shadmehr et al., 2010; Wolpert et al., 2011). These paradigms have proven very useful for understanding how the motor system adapts, but may be less ideally suited to investigating how the CNS learns an entirely new skill, or how motor performance is refined in absence of perturbations (Shmuelof et al., 2012). For instance, how does the brain initially learn the dynamics of the motor system? How does the CNS learn to refine and optimize control? Because BMIs create a new control system distinct from the natural, well-learned sensorimotor apparatus, it is uniquely suited to studying these questions. The ability to define novel transforms removed from the natural sensorimotor apparatus also opens the possibility to study neuroprosthetic skills and learning beyond the sensorimotor system.

## Studying learning with BMI systems

BMIs define novel functional circuits for action that actively engage subject learning and can be precisely manipulated in experiments. Recent studies in motor BMIs leveraged these unique properties to study skill learning and adaptation. Emerging work in afferent and efferent-afferent BMIs, as well as other interface systems also show the promise of BMI technology in learning studies.

Motor learning is thought to have distinct forms, including adaptation and skill formation, which may have different underlying neural mechanisms (Krakauer and Mazzoni, 2011). The same is likely true for BMI systems and abstract learning. While adaptation and skill can be separated in the natural motor system, these distinctions are currently less well-defined in BMI. Only a small number of studies have addressed learning in BMI, so information is limited, and few have attempted to model BMI learning processes. For the purposes of the proceeding discussion, we define “skill learning” as the process of learning to control a BMI system *de novo*, evidenced by the gradual formation of proficient performance. We use “adaptation” to refer to learning associated with compensating for perturbations to a previously-learned BMI system with proficient performance. Similarly, as noted above we use the term “neuroprosthetic skill” to refer to proficient BMI performance irrespective of physical movement. Such neuroprosthetic skills may be linked to more abstract forms of learning and skill. How our definitions of learning in BMI relate to adaptation and skill formation in the natural motor system, and the relationship of neuroprosthetic and abstract skills are fascinating and open questions we address in the discussion of future directions.

### Neuroprosthetic skill formation

There are clear behavioral signatures of skill—robust, reliable performance that can be rapidly recalled. But the neural representations underlying skill formation remain uncertain. The motor cortices appears to be involved in the formation and retention of motor memories and skills (Krakauer and Shadmehr, 2006), but what is the substrate of that memory? How the brain forms and stores memories is a critical question in motor learning, and neuroscience at large.

Ganguly and Carmena investigated this question by examining how subjects learned skilled BMI control (Ganguly and Carmena, 2009). Non-human primate subjects operated a closed-loop cursor BMI using single-unit activity from the motor cortex without overt arm movements. Critically, the mapping between cursor movement and neural activity—the “neuroprosthetic circuit”—was held constant for many days. Subjects became proficient in BMI control over days. The behavior showed many similarities to natural motor learning, with intra- and inter-session learning, and rapid recall of performance each day. Moreover, after achieving proficient control, a subject was able to learn a second decoder without disrupting the performance with the initial decoder. Together, these results suggest that BMI control was achieved via consolidation of a neuroprosthetic skill, identified by proficient, rapidly-recalled control of a disembodied actuator irrespective of natural movement.

The mapping between neural activity and output in BMI allows for thorough investigation of the neural underpinnings of neuroprosthetic skill. The direction tuning—relationship between firing rate and direction of target motion—of neurons contributing to the BMI decoder (“BMI neurons”) shifted as subjects improved their BMI performance. Direction tuning changed significantly early during learning, but became more stable as performance reached a plateau. Skill consolidation resulted in the formation of a stable neural “map” of the decoder that could be rapidly recalled. Changing BMI decoders daily disrupted skill and neural map formation, suggesting they are specifically tied to learning the input-output transform defined by the neuroprosthetic circuit (Ganguly and Carmena, 2009).This map was significantly different from that of natural arm movements. Yet, subjects could readily switch between arm and neuroprosthetic control, and neural activity showed corresponding rapid shifts between two different maps (Ganguly et al., 2011).

BMI also allows observation of brain areas not directly contributing to the task. What occurs in other parts of the motor cortex as a subject learns a neuroprosthetic skill? Examining the activity of neurons in motor cortex but not contributing to the decoded output during transform learning revealed large-scale changes in their firing properties and relation to the task (Ganguly et al., 2011). Non-BMI neurons’ preferred direction (cursor motion causing maximal firing) changed compared to arm movements, similar to BMI neurons. However, proficient neuroprosthetic control was associated with a reduction in non-BMI neurons’ modulation depths compared to BMI-neurons. Interestingly, the reduction in modulation depth was dependent upon the non-BMI neurons’ distance from BMI neurons. This effect was apparent in late, but not early, stages of learning, suggesting that it was linked to neuroprosthetic skill formation. Transform learning triggered a large-scale, reversible modification of the cortical network centered on the BMI neurons. Recent work in humans suggests that neuroprosthetic learning may also result in the formation of a more broadly distributed cortical network that extends well beyond the areas directly involved in control (Wander et al., 2013).

These results give tantalizing suggestions about the neural substrates of a skill. But where and how does this learning take place? Does neuroprosthetic learning involve similar brain structures as natural motor learning? To address these questions, Koralek et al. developed a BMI paradigm where rodents learned to control the pitch of an auditory cursor to reach one of two targets by modulating activity in primary motor cortex in the absence of physical movement (Koralek et al., 2012). They examined the activity of the dorsolateral striatum—a structure linked to motor skill learning—during learning of this abstract skill. Striatum neurons were modulated during neuroprosthetic control, and the activity in motor cortex—the output system—increased its coherence with the dorsolateral striatum as learning progressed. These coherence changes were also found to be specific to motor cortex neurons contributing to the decoder, consistent with the formation of a BMI-specific network (Koralek et al., 2013). Deletion of striatal N-methyl-aspartic acid (NMDA) receptors, which are necessary for corticostriatal long-term potentiation, severely impaired the development of this corticostriatal plasticity, and completely disrupted the subjects’ ability to learn neuroprosthetic skills. These results suggest that corticostriatal circuits are involved in learning skills, even when they do not require physical movement. Moreover, these results show that the process of transform learning not only elicits changes in motor cortical networks, but also recruits elements of the natural motor system outside of the cortex, such as the basal ganglia. Neuroprosthetic skill learning, then, may utilize the built-in mechanisms for natural motor learning.

Together, these studies give preliminary evidence for cortical substrates of learning and the involvement of deep-brain structures in their formation. They also demonstrate that BMIs provide a platform to study the formation of skills. Many questions remain—for instance, the physiological mechanisms driving cortical network formation are uncertain. The relationship between this neuroprosthetic skill, natural motor learning and abstract skills also remains to be fully explored. Expansions of this long-term transform learning paradigm can be used to further probe how this learning occurs.

### Adaptation in BMI

BMIs can also be used to study adaptation to carefully controlled perturbations. A recent series of studies took advantage of the ability to manipulate the decoder to probe the behavioral and neural mechanisms of adaptation learning in BMI (Jarosiewicz et al., 2008; Chase et al., 2012; Golub et al., 2012). Non-human primates controlled a cursor-based BMI driven by single-unit activity. After subjects achieved proficient control, the researchers perturbed the decoder by rotating the resulting cursor velocity for a given neural input, and examined if and how neural activity changed. This is akin to visuomotor rotations commonly used in motor learning (Krakauer et al., 2000). What’s more, they perturbed the input-output mapping of only a subset of units within the decoder to study if and how adaptation differed for perturbed and non-perturbed units. The behavioral responses to these decoder perturbations showed remarkable parallels to that of natural visuomotor rotations, with subjects initially producing curved trajectories that straighten over time. Removing the perturbation also revealed after-effects—curvature opposite that of the applied rotation—that quickly decayed.

The BMI paradigm allowed for careful examination of the adaptation strategies used by subjects and their neural correlates. While previous studies demonstrated shifts in motor cortex activity during natural motor adaptation, BMI provides knowledge of the precise mapping between neural activity and behavior, which can be used to more clearly interpret neural changes. There are several possible ways to solve a rotational perturbation task, including a global strategy of re-aiming to a new target, or local strategies to either reduce the contribution of perturbed units, or selectively rotating the action-directions of the perturbed units. Analysis revealed evidence for both global and local adaptation. Interestingly, global re-aiming dominated and the degree to which the different strategies were employed showed some dependence on the number of units perturbed. This suggests that there may be limits to the degree of neural adaptation, at least in the short time-frame of these experiments (Chase et al., 2012).

Behavioral manipulations in BMI control and clever analyses also allowed for quantification of the subjects’ control strategies and the time-scale of learning in this paradigm. Golub and colleagues removed visual feedback for the initial portion of reaches, and then used the timing of feedback corrections upon receiving visual feedback to quantify the control time-delay in BMI (Golub et al., 2012). This allowed the researchers to assess whether the subject based control operations on the perceived, delayed visual feedback or on estimates of the current cursor position, which would suggest the formation of an “internal model” of cursor movement. Their results suggest that subjects do indeed form internal predictions of cursor movement. Moreover, by analyzing the above-described decoder perturbation data, theses analyses also showed that learning may be accompanied by modification of this internal model and a method to quantify the time-scale of such adaptation.

By manipulating the decoding algorithm, these studies identified key neural components of learning and adaptation. However, additional work is needed to examine the underlying mechanisms. For instance, are changes in cortical firing driven by input to those areas or via synaptic plasticity? Are the different types of adaptation—global vs. local—achieved through the same or different means? What are the neural signatures of the “internal model” underlying BMI operation, and modified during adaptation? The ability to fully control the BMI system may prove extremely useful for answering these questions. Examination of non-BMI units in up-stream brain areas and within the same cortical area might shed light on the scale and specificity of adaptive mechanisms. Electrophysiology techniques could also be used to explore synaptic plasticity. The clear distinction between BMI and non-BMI units, and perturbed versus non-perturbed units will be essential for honing in on how physiological changes shape the circuit.

### Sensorimotor integration and sensory transform learning

The majority of learning-related BMI studies focus on efferent control. However, sensory BMIs can also be used to investigate key questions about sensorimotor learning and integration. Neural stimulation—via ICMS or optogenetic techniques—can be used to evoke percepts and influence cortical processing (Wilson et al., 1991; Romo et al., 2000; London et al., 2008; Weiland and Humayun, 2008; Tehovnik et al., 2009; Berg et al., 2013). Moreover, artificial neural stimulation can be integrated with natural sensory feedback to facilitate active-sensing tasks (O’Doherty et al., 2011; Venkatraman and Carmena, 2011). Emerging work also suggests that the integration of natural sensory information and artificial stimulation is modulated by the reliability of the sensory information (Dadarlat et al., 2012), in strong agreement with many observations of natural sensorimotor integration (Sabes, 2011). Together, these studies show that stimulation can convey useful information to subjects, making it possible to create closed-loop sensory BMI systems that operate parallel to the natural sensorimotor systems.

Interestingly, several studies suggest that the percepts evoked by artificial stimulation have only a slight similarity to their natural counterparts. For example, rats trained to respond to movement of the whiskers initially also responded to ICMS stimulation, but quickly learned to discern the two stimuli (Venkatraman and Carmena, 2011). Non-biomimetic approaches to neural stimulation have been shown to be effective (O’Doherty et al., 2011; Dadarlat et al., 2012). Thus, sensory BMIs also involve transform learning, where subjects learn a novel mapping between the artificially-evoked neural activity and environmental variables. However, studies of the underlying learning mechanisms involved in afferent BMIs are forthcoming. Closed-loop BMI studies have tremendous potential to illuminate the learning processes in sensory systems and the underlying neural mechanisms. Combining afferent and efferent BMIs (O’Doherty et al., 2011) is a similarly promising platform that may allow for careful study of how sensory inputs are transformed into actions.

### Beyond brain-interfacing: transform learning tools

The ability to create novel functional action circuits extends beyond BMI. Other signals from the CNS, such as muscle activity or limb kinematics, can be used to control artificially-defined systems. Myoelectric interfaces, for example, map electromyograms of select muscles to the motion of computer cursors via linear decoding algorithms (Radhakrishnan et al., 2008; Nazarpour et al., 2012). The joint angles of the human hand can also be artificially mapped to a cursor position (Mosier et al., 2005; Mussa-Ivaldi and Danziger, 2009; Liu et al., 2010). Much like BMI, these approaches create closed-loop control systems that are different from the natural sensorimotor system and can be used to investigate how the CNS learns to novel, abstract transforms. Subjects can readily learn to control these novel interfaces, even with arbitrary and non-intuitive mappings (Mosier et al., 2005; Radhakrishnan et al., 2008)

These two interface systems show tremendous potential for studying fundamental questions of motor learning and control. Myoelectric interfaces have been used to explore theories about muscle synergies and optimal control (Radhakrishnan et al., 2008; Nazarpour et al., 2012). Their work shows that subjects use coordinated patterns of muscle activity shaped to maximize task performance—hallmarks of optimal, synergy-based control—even in motor tasks disconnected from the natural sensorimotor apparatus. Kinematic interfaces have been used to explore how the motor systems deals with redundancy (Mosier et al., 2005; Mussa-Ivaldi and Danziger, 2009; Ranganathan et al., 2013). These mappings are highly redundant, and knowledge of the mapping’s structure allowed researchers to separate subjects’ movements into task-relevant and -irrelevant components. Transform learning was accompanied by significant reduction in task-irrelevant movements, suggesting that subjects learned to constrain their movements to those that contributed to cursor movements. Kinematic interfaces have also been used to study adaptation to perturbations, revealing previously unobserved distinctions between adaptation to different types of manipulations (Liu et al., 2010). The authors suggest that the extensive experience with reaching may influence motor adaptation and learning studies. Novel interfaces may provide new and critical insights into motor learning that cannot be readily assessed by studying the natural motor system.

Subjects operating BMIs, myoelectric-, and kinematic-interfaces all show clear hallmarks of skill learning. However, several have noted differences in the learning rates and strategies of these systems (Green and Kalaska, 2011; Jackson and Fetz, 2011; Chase et al., 2012). The fundamental differences in the control inputs, and their relation to the natural motor system, may be a key factor in these learning differences (Jackson and Fetz, 2011). The mechanisms underlying learning may depend significantly upon the type of inputs the user controls. Moreover, learning and adaptation in the natural motor system may involve multiple learning mechanisms given its highly distributed and hierarchical nature. Exploring learning in these different types of interfaces may help elucidate plasticity, learning, and adaptation mechanisms at different levels of the CNS.

The differences in learning across interface types also highlights the importance of fully understanding subject-models and instructions used in BMIs and the systems they define. A variety of animal models have been used in motor BMI studies—some allow subjects to move unrestricted (Gilja et al., 2012), while others block movement either via restraints (Jarosiewicz et al., 2008; Velliste et al., 2008) or temporary paralysis (Moritz and Fetz, 2011; Ethier et al., 2012). This has caused debate in the community, primarily focused on identifying models that best inform translation to paralyzed individuals (Nuyujukian et al., 2011). However, these different models may also have an impact on BMI learning studies. Consider a motor BMI where the subject is allowed to move their body unrestricted during control. While the BMI system only uses neural activity to control the actuator, the subject can adopt a kinematic-level learning strategy. That is, they may learn the task as a transform mapping between their body’s motion and the cursor, rather than learning the relationship between neural activity and cursor movement. Though both systems are interesting for studying learning, they may be solved in fundamentally different ways. Similarly, the context of BMI control, and its relationship to natural movement must also be considered. The presence of cognitive cues that distinguish BMI and natural movement contexts (e.g., movement restraints or removing the apparatus used for movement) could significantly shape learning strategies. Care must be taken to develop interface systems that are clearly defined.

## Future directions and conclusions

Use of BMI and other interface systems for studying learning is a nascent field. The latest developments clearly demonstrate their utility and potential. However, they only scratch the surface of many critical questions. There are many new avenues of exploration in BMI learning that have not yet been addressed.

### Relating BMI learning to motor and abstract learning

One critical question is how the BMI learning observed in these early studies relates to well-documented types of motor learning, such as adaptation and skill-formation (Krakauer and Mazzoni, 2011). These types of learning are thought to have different underlying mechanisms and neural implementations. Connecting BMI learning to these well-documented and modeled forms of learning could both facilitate better understanding of the neural mechanisms driving different types of learning, as well as more formal study of BMI learning. Here, we have defined skill as learning proficient BMI control *de novo*, and adaptation as compensating for perturbations to a well-learned BMI system with proficient control. The underlying learning mechanisms in these systems, however, may not be directly associated with adaptation and skill as defined in the natural motor system. For instance, are tuning changes that accompanied neuroprosthetic skill acquisition in Ganguly and Carmena (2009) solely a reflection of the neural representations of skill? Or does learning neuroprosthetic skill involve multiple learning mechanisms? Early learning could be driven by adaptation, where subjects modify existing neural patterns from their natural motor repertoire, reflected by tuning changes. Later refinements of control, however, might be more similar to skill formation, with increased precision of recruited neural activity patterns, consistent with the highly stable tuning maps observed in late learning.

Addressing these questions will require better understanding of *how* subjects learn BMI transforms. A particularly important question is if and how BMI learning is linked to natural movement. While BMIs can be controlled without overt movements, their relationship to movement and the natural system is unclear. Are BMIs controlled by repurposing existing motor repertoires, or via direct operant conditioning of neural activity to create new neural networks—or some combination? Understanding BMI’s relationship to the natural motor system will be critical for teasing apart the underlying mechanisms involved in BMI learning and relating them to those of natural motor learning. Better understanding this relationship is also crucial for relating neuroprosthetic skill (BMI learning irrespective of movement) to abstract skills.

Existing studies provide mixed evidence for how BMI control relates to the natural motor system. A recent study by Hwang et al. (2013) explored control strategies in a discrete-control BMI system driven by neural activity from the parietal reach region (PRR). The experimenters used the well-established visuomotor properties of PRR neurons to probe the strategy used to learn different decoders to select one of two possible actions. Their results suggested that subjects solved the task by aiming to alternate targets in order to select the desired target location. That is, BMI control relied explicitly on natural motor strategies. This strategy was further suggested by persistent activity of neurons not directly contributing to the target selection, which would not be expected by operant conditioning-based learning. Interestingly, these results differ substantially from studies with continuous-control BMIs, where long-term learning was accompanied by differential modulation of BMI units within motor cortex (Ganguly et al., 2011) and striatal interactions specific to BMI output neurons (Koralek et al., 2013). Similarly, decoder perturbation studies in motor cortices suggest that learning was not limited to global re-aiming strategies alone (Jarosiewicz et al., 2008; Chase et al., 2012). While these observations do not preclude the possibility that BMI learning is shaped by the natural motor repertoire, they strongly suggest that BMIs may create new neural networks rather than purely repurposing established ones. However, learning arbitrary decoders has been shown to require similar neural structures to that of natural motor learning, like the striatum (Koralek et al., 2012). BMI learning, then, may still leverage similar neural circuitry to that of the natural motor system.

Differences in learning across these studies highlights the need for careful consideration of the BMI system and task design, and how they may influence learning. These studies differed in both the brain areas used (PRR versus primary- and pre-motor cortex) and the types of feedback provided (discrete versus continuous), both of which may strongly influence learning. The type of feedback and subject instructions, in particular, can significantly influence learning in the natural motor system (Krakauer and Mazzoni, 2011; Taylor and Ivry, 2011). The type of feedback provided in BMI has also been shown to significantly impact learning, as rats were unable to learn a novel BMI task without continuous feedback (Koralek et al., 2012). If and how BMI learning is influenced by the brain area(s) used for control is also an open and interesting question—one that may be particularly important for understanding BMI learning’s relationship to abstract skills. Much like natural motor tasks, BMI learning may be shaped by system properties such as the control signals, feedback, and subject instructions. Careful manipulations of these properties may be particularly important to fully elucidate the mechanisms of BMI learning.

### Further open questions in BMI learning

The brain can learn arbitrary transforms, as evidenced by many demonstrations of non-biomimetic interface learning (Fetz, 1969, 2007; Radhakrishnan et al., 2008; Ganguly and Carmena, 2009; O’Doherty et al., 2011). The limits of such transform learning, however, are unclear. The majority of neurons in motor cortices can be modulated via biofeedback (Moritz and Fetz, 2011), but it is unknown if there are constraints on coordinated network activity that might limit transform learning. Exploring the relationship between BMI decoders and neural changes during learning may shed light on these questions. Does neural activity reach an “optimal” solution to a decoder, or do network dynamics limit adaptation? Similarly, little is known about how the structure of a transform influences learning. Are some decoder or encoder structures more readily learned? Does the relationship to natural representations matter? Is learning dependent on the neural ensembles and brain areas used for control? Exploring the relationship between transform structure, neural inputs, and learning might further elucidate how the CNS learns such mappings. This may also be particularly important for relating BMI learning to that of the natural motor system and abstract learning as noted above.

Combining BMI transform learning with established motor learning paradigms and physiological techniques will also better elucidate the neural substrates of skills. Interference and disruption of partially-consolidated motor memories is a well-studied and fascinating phenomenon (Krakauer and Shadmehr, 2006). What are the physiological mechanisms of skill consolidation and disruption? Neuroprosthetic skill learning shows clear parallels to motor learning and consolidation (Ganguly and Carmena, 2009; Ganguly et al., 2011). Exploring the physiological differences—for instance, synaptic modifications—in networks between consolidated and non-consolidated BMI decoders may help identify the mechanisms of stable memory formation. Finding causal interventions that can perturb skill consolidation will also be essential. It may also be possible to use neural stimulation to modify and shape neural network structure (Jackson et al., 2006) and study the resulting effects on skill consolidation.

BMIs create novel functional circuits for action that can be carefully manipulated to study the mechanisms of skill learning. Exploring learning in BMIs can make great contributions to our understanding of motor and abstract skill learning. What’s more, this knowledge may be particularly useful for developing rehabilitative and restorative therapies. For instance, understanding principles of transform learning and the underlying neural mechanisms may be particularly useful for developing rehabilitation strategies where subjects must relearn motor control (e.g., stroke). These insights will also help to design neuroprostheses that are easier to learn. Knowledge of the neural mechanisms of skill consolidation and disruption may also be essential for making BMIs that can be controlled in a variety of settings. The basic science and technological applications of BMI have a naturally symbiotic relationship.

## Conflict of interest statement

The authors declare that the research was conducted in the absence of any commercial or financial relationships that could be construed as a potential conflict of interest.

## Footnotes

^1^There are, however, notable exceptions of researchers that recognized the importance of closed-loop BMI learning and adaptation early on (e.g. Taylor et al., 2002; Wolpaw et al., 2002; Carmena et al., 2003). The EEG community has also recognized and integrated these aspects more readily (Wolpaw et al., 2002; Millán and Mouriño, 2003).
